# Mono or Dual Antiplatelet Therapy for Treating Patients with Peripheral Artery Disease after Lower Extremity Revascularization: A Systematic Review and Meta-Analysis

**DOI:** 10.3390/ph15050596

**Published:** 2022-05-12

**Authors:** Shang-Yu Tsai, Ying-Sheng Li, Che-Hsiung Lee, Shion-Wei Cha, Yao-Chang Wang, Ta-Wei Su, Sheng-Yueh Yu, Chi-Hsiao Yeh

**Affiliations:** 1 Department of Thoracic and Cardiovascular Surgery, Chang Gung Memorial Hospital, Linkou, Taoyuan 333, Taiwan; abc080063@gmail.com (S.-Y.T.); fishandchips0612@gmail.com (Y.-S.L.); taweisumd@gmail.com (T.-W.S.); yusy@cgmh.org.tw (S.-Y.Y.); 2 Department of Thoracic & Cardiovascular Surgery, Chang Gung Memorial Hospital, Keelung, Keelung 204, Taiwan; ycwang@cloud.cgmh.org.tw; 3 Department of Plastic and Reconstructive Surgery, New Taipei Municipal Tu-Cheng Hospital (Built and Operated by Chang Gung Medical Foundation), New Taipei City 236, Taiwan; ikemanleee@gmail.com; 4 Department of Plastic and Reconstructive Surgery, Chang Gung Memorial Hospital, Linkou, Taoyuan 333, Taiwan; 5 Department of General Surgery, Chang Gung Memorial Hospital, Keelung, Keelung 204, Taiwan; swchai1988@cgmh.org.tw; 6 College of Medicine, Chang Gung University, Taoyuan 333, Taiwan

**Keywords:** dual antiplatelet therapy, peripheral artery disease, randomized control trials, major adverse cardiac and cerebrovascular events, major adverse limb events

## Abstract

The efficacy of dual antiplatelet therapy (DAPT) for patients with peripheral artery disease (PAD) after lower-limb intervention remains controversial. Currently, the prescription of DAPT after an intervention is not fully recommended in guidelines due to limited evidence. This study compares and analyzes the prognosis for symptomatic PAD patients receiving DAPT versus monotherapy after lower-limb revascularization. Up to November 2021, PubMed/MEDLINE, Embase, and Cochrane databases were searched to identify studies reporting the efficacy, duration, and bleeding complications when either DAPT or monotherapy were used to treat PAD patients after revascularization. Three randomized controlled trials and seven nonrandomized controlled trials were included in our study. In total, 74,651 patients made up these ten studies. DAPT in PAD patients after intervention was associated with lower rates of all-cause mortality (HR = 0.86; 95% CI, 0.79–0.94; *p* < 0.01), major adverse limb events (HR = 0.60; 95% CI, 0.47–0.78; *p* < 0.01), and major amputation (HR = 0.78; 95% CI, 0.64–0.96) when follow-up was for more than 1-year. DAPT was not associated with major bleeding events when compared with monotherapy (OR = 1.22; 95% CI, 0.69–2.18; *p =* 0.50) but was associated with a higher rate of minor bleeding as a complication (OR = 2.54; 95% CI, 1.59–4.08; *p* < 0.01). More prospective randomized studies are needed to provide further solid evidence regarding the important issue of prescribing DAPT.

## 1. Introduction

Peripheral artery disease (PAD) is a chronic manifestation of atherosclerosis and affects the arteries of the lower extremities. The worldwide prevalence of PAD ranges from ~3% to ~12%, and the number of people with PAD increased by 23.5% globally from 2000 to 2010 [1]; furthermore, the critical limb ischemia (CLI) subset of these patients also increased, namely by about 2.9% from 2005 to 2009 [2]. The atherosclerotic characteristics associated with PAD have been found to share many common risk factors with coronary artery disease (CAD) and cerebrovascular disease (CVD) [3]. Thus, many PAD patients suffer from localized morbidities and systemic atherosclerotic complications, including CAD and CVD. In particular, among symptomatic PAD patients, their all-cause mortality appears to be significantly higher than that of patients without symptoms [4]. In a global review of the REACH registry [5], 39% of PAD patients were found to have concomitant CAD, while 10% had concomitant CVD, and 13% had all three comorbidities. A review article by Criqui et al. demonstrated that PAD patients had a 1.3 to 6.3-fold greater risk of a CAD-related death [6]. Previous studies have demonstrated that CAD and CVD result in 40% to 60% and 10% to 20% mortality in PAD patients, respectively [7]. Thus, the presence of a systemic atherosclerotic burden may cause various diseases, and thus result in major problems, which in turn lead to increased mortality among PAD patients [8].

Therefore, treatments that focus on decreasing mortality due to systemic atherosclerotic diseases might also effectively reduce the mortality of PAD patients.

Apart from the introduction of aggressive lifestyle modifications that reduce overall cardiovascular risk [9], the optimal medical treatment for these diseases remains risk-factor control, and this should also be beneficial to any cardiovascular diseases present in symptomatic PAD patients by preventing deterioration. Revascularization interventions have been recommended for symptomatic PAD patients presenting with intermittent claudication (IC) or critical limb ischemia (CLI; this relieves pain, prevents major adverse limb events (MALEs), and reduces the overall cost of healthcare [10,11]. Long-term antiplatelet monotherapy is recommended to help prevent systemic atherosclerosis progression and to reduce major adverse cardiac and cerebrovascular events (MACCEs) among symptomatic PAD patients [12,13]. Dual antiplatelet therapy (DAPT) has been proved to reduce MACCEs in CAD patients who are undergoing percutaneous coronary intervention [14]. Whether DAPT is able to diminish MACCEs among symptomatic PAD patients who are receiving various interventions, including surgical bypass and endovascular procedures with or without stent placement, remains unknown. Currently, the prescription of DAPT for symptomatic PAD patients after intervention is not fully recommended in available guidelines [13,15] due to there being limited evidence regarding a beneficial effect in terms of a reduction in MACCEs and MALEs. One concern related to the prescription of DAPT in this context is the possible risk of major bleeding complications affecting these symptomatic PAD patients.

This study focuses on comparing and analyzing the prognosis of symptomatic PAD patients who are receiving DAPT after intervention, and this is compared with monotherapy after intervention. Our results, which are based on a systematic review and a meta-analysis, offer further solid evidence that should help to guide the use of postinterventional antiplatelet therapy.

## 2. Materials and Methods

This study was conducted using strict adherence to the recommendations of PRISMA (Preferred Reporting Items for Systematic Reviews and Meta-Analyses) and has been registered at PROSPERO (ID: CRD42021258446) (The date of registration: 21 October, 2021) [16].

### 2.1. Search Sources and Strategy

We searched various databases, namely the PubMed/MEDLINE, Embase, and Cochrane databases, in order to identify studies that report the efficacy, duration, and bleeding complications related to DAPT or monotherapy among PAD patients after revascularization procedures. The literature search strategy included both MeSH terms and free text related to PAD. The subject heading terms “(dual antiplatelet therapy) AND (peripheral artery disease) AND ((endovascular treatment) OR (percutaneous transluminal angioplasty) OR (revascularization) OR (bypass))” were used for the search. Additional articles were identified by reviewing the references included in any of the identified studies.

A dataset was created and extracted by two of the authors (S.-Y.T. and Y.-S.L.). Abstracts, full-text articles, patient characteristics of interest, and relevant outcomes were retrieved where relevant. All English-language studies reported before November 2021 were included. The risk of bias was assessed using the Cochrane collaboration’s revised tool for assessing the risk of bias in randomized trials (RoB 2.0) [17], as well as the risk of bias in nonrandomized studies–of interventions (ROBINS-I) tool [18]. All randomized control trials (RCTs) and retrospective nonrandomized controlled trials (NRCTs) were included. Case reports and review articles were excluded.

### 2.2. Study Selection and Data Extraction

Patients: Symptomatic PAD patients, who presented with IC or CLI and underwent surgical bypass or endovascular intervention, were included. Intervention and comparison: After the intervention, patients must have undertaken either monotherapy (aspirin or clopidogrel) or DAPT (defined as aspirin plus any P2Y12 receptor antagonist, including clopidogrel, ticagrelor, prasugrel, or ticlopidine). The treatment duration of DAPT after intervention needed to be more than 1 month. Surgical venous/prosthetic graft implantation is defined here as arterial bypass surgery. Endovascular therapy is defined as including balloon angioplasty with or without stenting. The median postoperative follow-up duration must be more than 1 year.

### 2.3. Outcomes

The primary outcome was all-cause mortality. The secondary outcomes were MACCEs, including myocardial infarction (MI), stroke (whether ischemic or hemorrhage), and major adverse limb events (MALEs), including major amputation (above the ankle), amputation-free survival, and target lesion revascularization (TLR) (whether surgical or endovascular). Other safety endpoints, namely major and minor bleeding, were included based on the bleeding risk definitions [19,20,21] outlined in this study (Supplementary Table S1). Various subgroup analyses, such as RCT vs. NRCT and endovascular vs. surgical bypass, were also performed.

### 2.4. Assessment of Methodological Quality

The risk of bias related to the included articles was assessed by three authors (S.-Y.T., Y.-S.L., and C.-H.Y.) according to RoB 2.0 for RCTs and the ROBINS-I tool for NRCTs.

### 2.5. Data Synthesis and Analysis

The hazard ratio (HR) with corresponding 95% confidence intervals (CIs) of the primary and secondary outcomes were retrieved and calculated across the relevant studies. When HRs were not reported in those studies, we sent an e-mail asking for the raw data or any relevant HR data in order to carry out further analysis. If the authors did not reply or did not offer the relevant information, we extracted the data from Kaplan–Meier curves published in the included articles using DigitizeIt, version 2.5 (http://www.digitizeit.de/, accessed on 1 December 2021) (I. Bormann, Germany) software; this allows the extraction of data from a graphical image, and each HR was derived by a “curve approach” as described by Tierney et al. [22]. The random-effects model was used for meta-analysis due to the broad range of different surgical interventions used, the different degrees of the disease severity, and other sources of variance. For safety outcome analysis, we used the odds ratio (OR) for the meta-analysis with the corresponding 95% CIs. Statistical heterogeneity was assessed using a cut-off value of *p* ≤ 0.10 for the Cochrane Q test results or I^2^ ≥ 50% for statistical heterogeneity. Potential publication bias was estimated by plotting effect size, and this was analyzed with funnel plots. All analyses were performed using R (version 3.6.1) (R Foundation for Statistical Computing, Vienna, Austria).

## 3. Results

### 3.1. Studies and Patient Characteristics

**Figure 1:**
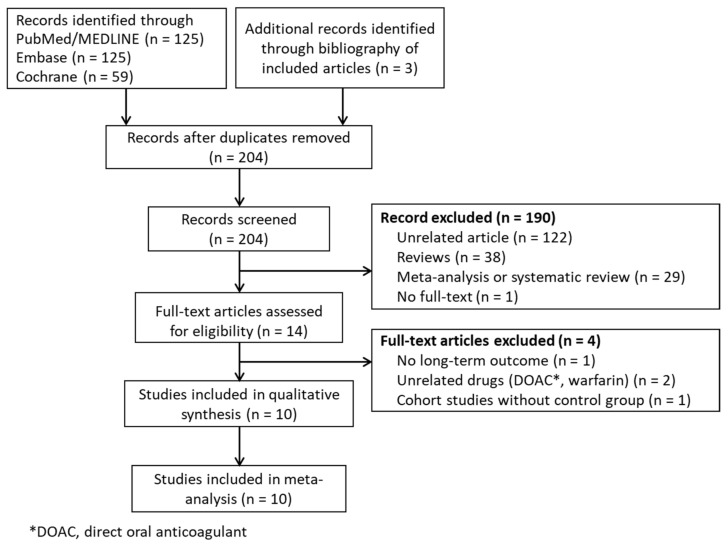
PRISMA flow diagram.

In total, there were 312 potential studies identified by screening the three databases (Figure 1). Three additional studies were identified by reviewing the bibliographies of the identified articles. By accessing the article abstracts and titles, 190 studies were excluded due to irrelevance or because they were review articles. One article was excluded due to the fact that the full text was unable to be found. In total, 14 full-text articles were retrieved and used. After careful analysis of these articles, one study without any long-term outcomes, two studies where control groups were absent, and one study designed to compare unrelated drugs, were excluded at the final stage. Finally, ten full-text articles, including three RCTs [23,24,25], three NRCTs with propensity-score-weighted matching (PSM) [26,27,28], and four NRCTs [29,30,31,32], were retrieved for quantitative synthesis.

A total 74,651 patients from the above ten reports were included in our study. Of these patients, 42,692 (57.2%) and 31,959 (42.7%) patients received DAPT and monotherapy, respectively. The majority of patients were men (63.4%), and most patients had received endovascular intervention (60.2%). In the included articles, the prevalence of CAD was up to 31.8%, CVD was about 14.7%, and DM was 45.5%. More than 95% of the patients in the included studies took aspirin (75–100 mg/day) plus clopidogrel (75 mg/day), as DAPT and the duration of treatment ranged from more than 1 month to more than 1 year after lower-limb intervention. The detailed study designs and characteristics of patients from the included studies are shown in Table 1. The DAPT regimens and complications of included studies are demonstrated in Supplementary Table S2.

### 3.2. Risk of Bias

The risk of bias within the described findings for RCTs (Supplementary Figure S1) and NRCTs (Supplementary Figure S2) was assessed by RoB 2.0 and ROBINS-I. The overall risk of bias was low in two of the three RCTs [23,25]. Bias due to deviations from the intended interventions was of some concern in one RCT [24] due to some patients (about 4%) undergoing crossover from the control group to the treatment group. In the four NRCTs, the use of an allocation method other than randomization means that these groups were unlikely to be comparable, which resulted in increased selection bias; three of the articles performed PSM to reduce treatment selection bias and adjust for confounding variables related to NRCTs [33]. The overall risk of bias was low in one NRCT [26], moderate in five NRCTs [27,28,29,30,31], and serious in one NRCT [32]. Publication bias was analyzed using funnel plots, and this revealed no obvious asymmetry regarding each investigated outcome (Supplementary Figure S3).

### 3.3. All-Cause Mortality

**Figure 2:**
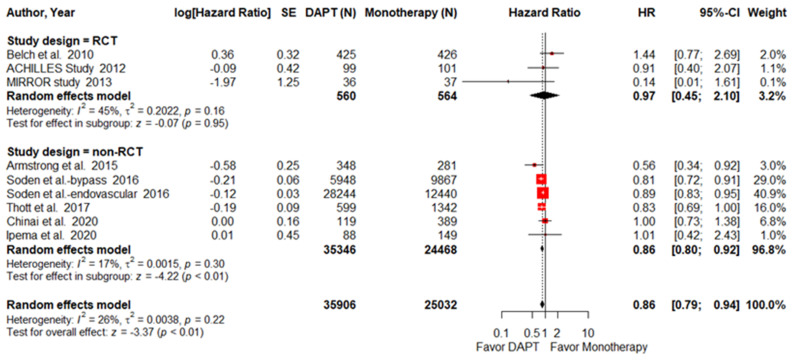
Forest plot of all-cause mortality. DAPT significantly reduced all-cause mortality for PAD patients after lower-limb revascularization. HR, hazard ratio; CI, confidence interval; DAPT, dual antiplatelet therapy; SE, standard error; RCT, randomized control trial. Adapted from refs. [23,24,25,26,27,28,29,30,31].

Eight articles, including 3 RCTs and 5 NRTCs, were pooled into the analysis to assess all-cause mortality. The pooled HR of all-cause mortality was significantly lower in PAD patients treated with DAPT (HR = 0.86; 95% CI, 0.79–0.94; *p* < 0.01; I^2^ = 25.7%; Figure 2). Interestingly, the survival benefit of DAPT was sustained in the NRCT subgroup analysis (HR = 0.86; 95% CI, 0.80–0.92; *p* < 0.01; I^2^ = 17.4%) but disappeared in the RCT subgroup analysis (HR = 0.97; 95% CI, 0.45–2.10; *p* = 0.95 I^2^ = 45.3%). The surgical method subgroup analysis showed that PAD patients treated with DAPT had a lower mortality rate after they had received endovascular intervention (HR = 0.87; 95% CI, 0.80–0.95; *p* < 0.01; I^2^ = 8.4%, Supplementary Figure S4). However, DAPT did not reduce mortality among the PAD patients who had received lower-limb bypass surgery (HR = 0.99; 95% CI, 0.58–1.70; *p* = 0.98; I^2^ = 68.47%).

#### 3.3.1. MACCEs

MACCEs were reported in four articles, consisting of one RCT and three NRCTs. DAPT reduced MACCEs in PAD patients after they had undergone a revascularization procedure (HR = 0.80; 95% CI, 0.52–1.24; *p* = 0.32; I^2^ = 68%, Supplementary Figure S5A), but this was not statistically significant and there was a high level of heterogeneity. There was no significant different in the occurrence of MACCEs in patients from either the RCT analysis (HR = 1.09; 95% CI, 0.65–1.82; *p* = 0.74) or in patients from the NRCT analysis (HR = 0.72; 95% CI, 0.44–1.20; *p* = 0.21; I^2^ = 69%) studies.

#### 3.3.2. MI

DAPT did not reduce MI in PAD patients after they had undergone a revascularization procedure (HR = 1.15; 95% CI, 0.54–2.45; *p* = 0.72; I^2^ = 44.3%, Supplementary Figure S5B). Similar results are also found for the RCT (HR = 0.81; 95% CI, 0.32–2.06; *p* = 0.66) and NRCT (HR = 1.59; 95% CI, 0.39- 6.50; *p* = 0.52; I^2^ = 68%) studies.

#### 3.3.3. Stroke

Strokes were recorded in three studies. DAPT treatment of PAD patients after revascularization, compared with patients receiving monotherapy antiplatelet treatment, did not reduce the occurrence of stroke (HR = 1.11; 95% CI, 0.59–2.10; *p =* 0.74; I^2^ = 0%, Supplementary Figure S5C). Furthermore, there was no significant difference in the occurrence of strokes among the patients included in either the RCT (HR = 1.02; 95% CI, 0.41–2.55; *p =* 0.97) or NRCT (HR = 1.21; 95% CI, 0.50–2.91; *p =* 0.68; I^2^ = 0%) studies.

### 3.4. Major Adverse Limb Events

**Figure 3:**
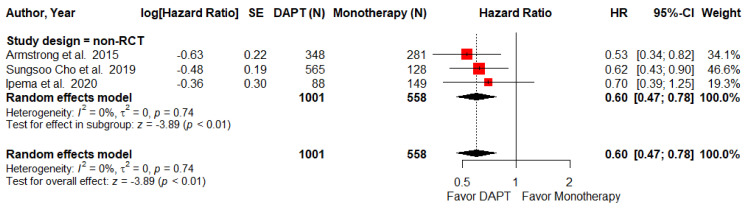
Forest plot of MALEs. DAPT significantly reduced the MALEs for PAD patients after lower-limb revascularization in the non-RCT subgroup. HR, hazard ratio; CI, confidence interval; DAPT, dual antiplatelet therapy; MALEs, major adverse limb events; SE, standard error; RCT, randomized control trial. Adapted from refs. [26,28,31].

Only three NRCTs endovascular intervention studies provided data regarding MALEs (defined as lower-limb ischemia causing major amputation, loss of patency, or re-intervention at the target lesion). The pooled result demonstrates that DAPT after endovascular intervention in PAD patients significantly reduced the risk of MALEs (HR = 0.60; 95% CI, 0.47–0.78; *p* < 0.01; I^2^ =0%, Figure 3).

#### 3.4.1. Major Amputation

**Figure 4:**
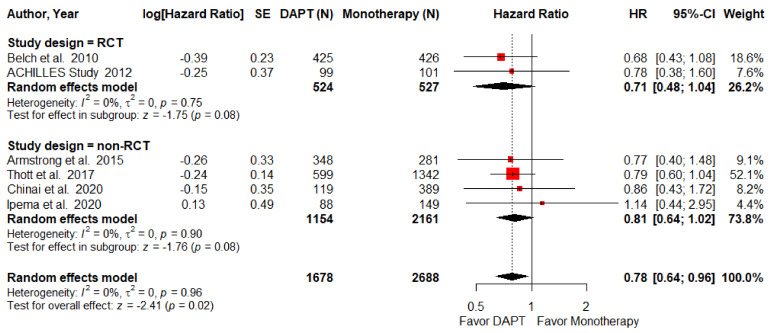
Forest plot of major amputation. DAPT significantly reduced major amputation for PAD patients after lower-limb revascularization. HR, hazard ratio; CI, confidence interval; DAPT, dual antiplatelet therapy; SE, standard error; RCT, randomized control trial. Adapted from refs. [23,24,26,29,30,31].

Major amputation rate was defined as amputation above the ankle within three years. Five studies, including 1 RCTs and 4 NRCTS, made up this part of the analysis. The major amputation rate was found to be significantly lower among the DAPT group (HR = 0.78; 95% CI, 0.64–0.96; *p =* 0.02; I^2^ = 0%, Figure 4). However, this result was not evident independently for either the RCT (HR = 0.68; 95% CI, 0.43–1.08; *p =* 0.1) or the NRCT (HR = 0.81; 95% CI, 0.64–1.02; *p =* 0.08; I^2^ = 0%) subgroup analyses.

#### 3.4.2. Re-Intervention

Six studies, including 3 RCTs and 3 NRCTS, were pooled to carry out the analysis of reintervention. The median follow-up period in these six papers was about two years (ranging between one to three years). There was no difference between DAPT and monotherapy (HR = 0.94; 95% CI, 0.85–1.04; *p =* 0.24; I^2^ = 0%, Supplementary Figure S5E). When subgroup analysis was carried out for re-intervention and when DAPT and monotherapy were compared, the results using the RCTs (HR = 0.83; 95% CI, 0.63- 1.10; *p =* 0.19; I^2^ = 0%) and the NRCTs (HR = 0.85; 95% CI, 0.61–1.17; *p =* 0.31; I^2^ = 34%) were similar. However, DAPT did seem to significantly reduce the re-intervention rate among PAD patients who received endovascular intervention (HR = 0.63; 95% CI, 0.43–0.93; *p =* 0.02; I^2^ = 0%).

### 3.5. Bleeding Complications

**Figure 5:**
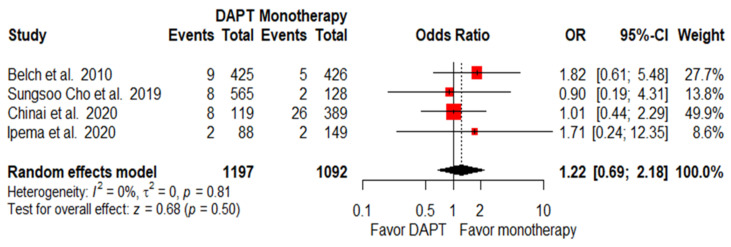
Forest plot of major bleeding. PAD patients after lower-limb revascularization with DAPT had a similar major bleeding rate as those with monotherapy. OR, odds ratio; CI, confidence interval; DAPT, dual antiplatelet therapy. Adapted from refs. [23,28,30,31].

Only four articles reported information on major bleeding complications (defined in Supplementary Table S1) after antiplatelet therapy among PAD patients who had received lower-limb interventions. There was no significant difference regarding major bleeding complications after lower-limb revascularization between the patients who were treated with DAPT and those who underwent monotherapy antiplatelet treatment (OR = 1.22; 95% CI, 0.69–2.18; *p* = 0.50; I^2^ = 0%, Figure 5). Three articles reported minor bleeding complications (defined in Supplementary Table S1). Patients in the DAPT group had a significantly higher frequency of minor bleeding compared with patients who underwent monotherapy (OR = 2.54; 95% CI, 1.59–4.08; *p* < 0.01; I^2^ = 0%, Supplementary Figure S6).

## 4. Discussion

In the study, we provided evidence of the beneficial effects of DAPT among PAD patients who had undergone lower-limb revascularization. Four main findings are able to be pinpointed in this study. Firstly, DAPT significantly reduces the all-cause mortality for PAD patients after lower-limb revascularization. Secondly, DAPT significantly reduces major amputation among PAD patients after lower-limb revascularization. In the subgroup analysis from the NRCTs, DAPT was also found to be beneficial in that it reduced MALEs. Thirdly, PAD patients who underwent DAPT had a similar major bleeding rate to those who underwent monotherapy. However, a higher minor bleeding rate was noted among PAD patients who received DAPT. Finally, In the subgroup analysis, PAD patients who received endovascular intervention with DAPT showed a significant reduction in the all-cause mortality rate (HR = 0.87; 95% CI, 0.80–0.95; *p* < 0.01; I^2^ = 8.4%), in MALEs (HR = 0.60; 95% CI, 0.47–0.78; *p* < 0.01; I^2^ =0%), and in the need for re-intervention (HR = 0.63; 95% CI, 0.43–0.93; *p =* 0.02; I^2^ = 0%).

### 4.1. Controversy about DAPT on Reduction in MACCEs

There has been up to the present no large-scale meta-analysis that has pooled multiple RCT and NRCT data in order to provide evidence about antiplatelet treatment of symptomatic PAD patients after they had undergone lower-limb revascularization. The beneficial effects of DAPT, whereby it reduces all-cause mortality, has only been reported by Navarese et al. [34]. Our results provide important evidence that DAPT is able to reduce all-cause mortality among PAD patients after lower-limb revascularization. Among PAD patients who received endovascular intervention, DAPT also significantly reduced all-cause mortality. PAD is a marker of a systemic atherosclerotic burden and shares many risk factors with the development, outcome, and prevention of cerebrovascular and coronary atherosclerosis [3]. CAD and CVD are the most common causes of death for PAD patients. However, the question of whether DAPT should be prescribed to PAD patients after lower-limb revascularization has remained unanswered. The major guidelines, including the 2016 ACC/AHA PAD guidelines (class IIb, level of evidence B) [13] and the 2017 ESC/ESVS lower-extremity artery disease guidelines (class IIa, level of evidence C) [35], do not fully recommend DAPT due to a lack of strong evidence. However, these guidelines recommend DAPT for reducing the risk of MACCEs, even though the effectiveness of DAPT has not been well-established in this area. Although our results show that DAPT has a tendency to reduce MACCEs, our findings were that this was without statistical significance. Furthermore, there was a high degree of heterogeneity (I^2^ = 69%, *p =* 0.04) within the pooled studies used in the MACCEs analysis. Two studies among them showed non-significantly harmful effects regarding MACCE reduction, while one retrospective study from Ipema et al. [31] demonstrated a 50% increase in MACCEs among the DAPT group. However, the study from Ipema et al. excluded patients with complicated lower-limb occlusion among whom interventions were either unsuccessful or there was re-intervention. As a consequence, the study from Ipema et al. had a higher standard error and was clearly subject to selection bias. After excluding the Ipema et al. study, DAPT still brought about no significant reduction in the risk of MACCEs (HR = 0.69; 95% CI, 0.46–1.03; *p* = 0.07) (Supplementary Figure S7), and there was still a high degree of heterogeneity (I^2^ = 61%, *p =* 0.08) among the remaining pooled studies. Another RCT from Belch et al. [23] also demonstrated an 8% increase in MACCEs among the DAPT group. However, the definition of a MACCE in Belch et al. is different from the other pooled studies (Supplementary Table S1), and this was probably the cause of the high heterogeneity. Therefore, at the present time, we cannot conclude that DAPT is able to reduce MACCEs and therefore has beneficial effects, although the HR does seem to be lower among the DAPT group.

### 4.2. DAPT on MALEs Reduction

A lack of high-quality evidence also hinders deciding whether DAPT should be recommended for PAD patients after lower-limb revascularization in order to bring about a reduction in MALEs [13,35]. A previous study from Hess et al. indicated that 12.9% of PAD patients undergoing peripheral revascularization were re-hospitalized due to a MALE [36]. They also found that these patients had an increased risk of suffering from subsequent cardiovascular and limb events (HR: 1.34; 95% CI 1.28–1.40) as well as being subject to major amputation or peripheral revascularization (HR 8.13; 95% CI 7.96–8.29). Sonia et al. also published similar results, whereby PAD patients with MALEs had a higher risk of subsequent hospitalization (HR 7.21; *p* < 0.0001), subsequent amputation (HR: 197.5; *p* < 0.0001), and death (HR: 3.23; *p* < 0.001) [37]. Consequently, any reduction in MALEs might be associated with a better prognosis for PAD patients. In our study, DAPT is significantly beneficial in that it prevents the occurrence of MALEs with a 40% reduction in risk. Hence, the present analysis indicates that DAPT after lower-limb revascularization is able to reduce the risk of mortality and prevent the occurrence of a range of limb-related events. The multicenter registry database in the United States shows that fewer than 50% of PAD patients, who were not receiving baseline DAPT, receive DAPT after lower-limb revascularization [38].

### 4.3. Safety Concerns about DAPT

In terms of safety concerns, although DAPT does not increase the risk of major bleeding, our results indicate that DAPT is associated with a higher risk of minor bleeding. In the study of CAD patients who had received the percutaneous coronary intervention (PCI), DAPT, compared with monotherapy, did not increase the risk of major bleeding (RR 1.12; 95% CI 0.70–1.78; *p* = 0.64) but did significantly raise the risk of minor bleeding (RR 1.68; 95% CI 1.06–2.68; *p* = 0.03) [14]. A similar result is also found for PAD patients who did not receive DAPT [39]. Based on our findings, DAPT is relatively safe and can be used safely for PAD patients after lower-limb revascularization.

### 4.4. Protective Effects of DAPT on Different Revascularization Procedures

There has been increasing attention paid to PAD, with the total number of lower-limb revascularization almost doubling over the decade from 1996 to 2006 [40]. As endovascular techniques and devices have improved, the number of patients per year who underwent endovascular intervention increased by about threefold, with a 40% decrease in surgical bypass procedures per year between 1999 and 2007 [41]. Our findings prove that DAPT is able to reduce the mortality and re-intervention rate compared with monotherapy among PAD patients after endovascular intervention, but this is not true for the surgical bypass group. The reason for this is unclear, but some papers have suggested it is related to the type of surgical grafts implanted. Some studies have revealed that DAPT seems to be only beneficial when PAD patients receive a prosthetic graft bypass, and this is associated with an increased prosthetic graft patency rate and a reduction in the amputation rate [23,32]. On the other hand, another study has shown that patients who receive vein grafts with anticoagulation therapy benefit from a lower rate of graft occlusion [42]. Additional studies are needed that investigates the best treatment for PAD patients who undergo a surgical bypass procedure.

### 4.5. Duration of Antiplatelet Treatment

In our systematic review, we have found that the duration of DAPT was very different between the various pooled studies; the time course of the interventions varied from 1 to 24 months. The optimal duration of antiplatelet treatment for PAD patients after lower-limb revascularization thus remains unknown. Two retrospective studies [43,44] revealed that a duration of DAPT treatment of 3 months and 12 months did not show a favorable outcome after the 12-month follow-up. However, Cho et al. have shown that DAPT for more than 6 months does result in a significantly lower risk of MACCEs and MALEs [28]. For CAD patients after PCI, DAPT is generally prescribed for at least 12 months in order to prevent ischemic events (class I, level of evidence B) [45,46]. Another study also has shown that short-term DAPT, compared with prolonged DAPT, reduces major bleeding complications but increases the risk of MI [47]. Two ongoing RCTs: ASPIRE-PAD (https://ClinicalTrials.gov/show/NCT02217501, accessed on 10 January 2022) and LONGDAPTPAD (https://ClinicalTrials.gov/show/NCT02798913, accessed on 10 January 2022) are investigating whether prolonged DAPT treatment, namely more than 12 months, might lead to better outcomes without increasing the bleeding risk. Future studies are needed to identify the optimal duration of DAPT when treating PAD patients after lower-limb intervention.

### 4.6. Novel Dual Pathway Regimen on PAD Patients

Recently, many studies have demonstrated that a novel dual pathway regimen (anti-coagulation plus aspirin) is more beneficial than monotherapy among PAD patients. The COMPASS trial reported that the novel dual pathway regimen significantly lowered the incidence of MACCEs among chronic PAD patients [37]. The VOYAGER-PAD trials revealed that the novel dual pathway regimen was able to reduce the risk of acute limb ischemia (HR 0.67; 95% CI 0.55–0.82) among PAD patients after lower-extremity intervention [48]. However, the VOYAGER-PAD trial also showed no significant reduction in all-cause mortality (HR 1.08; 95% CI 0.92–1.27; *p* = 0.34) and major amputation (HR 0.89; 95% CI 0.68–1.16). The ePAD trial, a head-to-head RCT to compare direct oral anticoagulant plus aspirin with DAPT among PAD patients undergoing lower-limb endovascular therapy, showed no significant benefit regarding a reduction in the incidence of limb-related events (RR 0.82; 95% CI 0.56–1.18; *p* = 0.3) [49]. As a consequence, more evidence is needed in order to clearly prove that the novel dual pathway regimen is better than DAPT among PAD patients after lower-limb revascularization.

### 4.7. Limitations

There are several limitations to our study. The NRCTs are limited by an unavoidable additional risk of bias and provide less precise information compared with the RCTs. Although it cannot replace a true RCT, the PSM method does help to minimize confounding factors and reduce any selection bias. Therefore, we believe that our conclusions are justifiable and generalizable. The second limitation involves the pooled forest plot used as part of the meta-analysis of RCTs and NRCTs; this may have concerns regarding the overall effect. Another limitation is that the research objects in the included studies are mostly related to western countries. The application of the study results, which were obtained using Caucasian populations, to other ethnic populations is questionable. Asian populations have a higher level of clopidogrel resistance [50,51,52]. In our pooled studies, most of the combinations used for the DAPTs involve clopidogrel plus aspirin. However, antiplatelet studies of PAD patients after revascularization involving Asian populations are very rare. Therefore, the transfer of the findings of currently available studies to the other race groups needs to be performed with great care.

## 5. Conclusions

DAPT, compared with monotherapy, is able to reduce all-cause mortality, reduce the occurrence of MALEs, and reduce amputation among PAD patients after lower-extremity revascularization; this occurs without an increased risk of major bleeding. In addition, the revascularization method within the endovascular subgroup significantly affects the mortality rate, the occurrence of MALEs, and the re-intervention rate when the patient is treated with DAPT after the intervention. Therefore, DAPT should be prescribed to PAD patients after lower extremity revascularization without worrying about major bleeding.

## Figures and Tables

**Table 1:** Characteristics and results of the included studies.

Study	Type	Patients (n)	Setting	Intervention (%)	DAPT Therapy	DAPT Duration (Months)	Monotherapy	Follow-Up (Months)	Outcomes
Belch et al. 2010 [23]	Prospective multicenter RCT	951	Atherosclerotic PAD	Bypass surgery (100)	Asp and Clo	6–24	Aspirin	6–24	Graft occlusion, revascularization, amputation or death
ACHILLES study 2012 [24]	Prospective multicenter RCT	200	PAD, Rutherford class 3 to 5	PTA only (50.5) or PTA plus stenting (49.5)	Asp and Clo or Ticl	6	Aspirin	12	Primary patency and binary restenosis
MIRROR study 2013 [25]	Prospective RCT	80	PAD, Rutherford class 3 to 5	PTA only (37.5) or PTA plus stenting (62.5)	Asp and Clo	6	Aspirin	12	Platelet activation marker and all-cause mortality
Armstrong et al. 2015 [26]	PSM retrospective study	629	PAD, Rutherford class 1 to 6	PTA only (80)	Asp and Clo (98.3%), Ticl (0.3%), or Pra (1.4%)	≥6	Asp	36	MACCEs
Soden et al. 2016 [27]	PSM retrospective study	15,985	PAD, Rutherford class 0 to 6 or ALI	Bypass surgery (100)	Asp and Clo, Pra, Ticl or Tica	≥12	Asp	12–60	All-cause mortality
Soden et al. 2016 [27]	PSM retrospective study	40,684	PAD, Rutherford class 0 to 6 or ALI	Endovascular (100)	Asp and Clo, Pra, Ticl or Tica	≥12	Asp	12–60	All-cause mortality
Thott et al. 2017 [29]	Retrospective study	1941	PAD, Rutherford class 4 to 6	PTA only (58), PTA and stenting (33), and subintimal angioplasty (9)	Asp and Clo	≥1	Aspirin	24	Major amputation or mortality
Cho et al. 2019 [28]	PSM retrospective study	693	PAD, Rutherford class 1 to 6	Endovascular (100)	Asp and Clo	1–12	Asp or Clo	40	Major amputation or mortality
Chinai et al. 2020 [30]	Retrospective study	508	PAD, Rutherford class 4 to 6	PTA only (61.2) or PTA plus stenting (38.8)	Asp and Clo (95.0%), Pra (4.2%), or Tica (0.8%)	3	Asp or Clo	36	Amputation-free survival
Ipema et al. 2020 [31]	Retrospective study	237	PAD, Rutherford class 1 to 6	PTA only (45.3) or PTA plus stenting (54.7)	Asp and Clo	3–12	Asp or Clo	12	MALEs
Belkin et al. 2021 [32]	Retrospective study	13,020	PAD, Rutherford class 0 to 6	Bypass surgery (100)	Asp and Clo (97.7%), Pra (1%), Tica (0.8%), or other (0.5%)	12	Asp	9–21	Patency of bypass graft

PSM, propensity-score-weighted matching; PAD, peripheral artery disease; PTA, percutaneous transluminal angioplasty; DAPT, dual antiplatelet therapy; MACCEs, major adverse cardiac and cerebrovascular events; MALEs, major adverse limb events; ALI, acute limb ischemia; Asp, aspirin; Clo, clopidogrel; Pra, prasugrel; Tica, ticagrelor; Ticl, ticlopidine.

## Data Availability

Data is contained within the article and Supplementary Materials.

## Supplementary Materials

The following supporting information can be downloaded at: https://www.mdpi.com/article/10.3390/ph15050596/s1, Figure S1: Bias analysis for RCTs with RoB 2.0; Figure S2: Bias analysis for the NRCTs using ROBINS-I; Figure S3: Funnel plot analysis assessing publication bias; Figure S4: Forest plots of the operation-method subgroup meta-analysis used for comparison between DAPT and monotherapy among PAD patients after lower-limb revascularization; Figure S5: Forest plots of the study-design subgroup meta-analysis used for comparison between DAPT and monotherapy in PAD patients after lower-limb revascularization; Figure S6: Forest plots of the minor bleeding meta-analysis used for comparison between DAPT and monotherapy among PAD patients after lower-limb revascularization; Figure S7: Forest plots of the major adverse cardiac and cerebrovascular events meta-analysis used for comparison between DAPT and monotherapy after exclusion of the article by Ipema et al.; Table S1: Definition of the outcomes; Table S2: Dosage of DAPT and complications of the included studies; Table S3: PRISMA checklist.

## Abbreviations

ACC	American college of cardiology
AHA	American heart association
AIOD	Aortoiliac occlusive disease
CI	Confidence intervals
CLI	Critical limb ischemia
CAD	Coronary artery disease
CVD	Cerebrovascular disease
DAPT	Dual antiplatelet therapy
DM	Diabetic mellitus
DOAC	Direct oral anticoagulant
ESC	European society of cardiology
ESVS	European society for vascular surgery
HR	Hazard ratio
IC	Intermittent claudication
MACCEs	Major adverse cardiac and cerebrovascular events
MALEs	Major adverse limb events
MI	Myocardial infarction
NRCTs	Nonrandomized controlled trials
OR	Odd ratio
PAD	Peripheral artery disease
PCI	Percutaneous coronary intervention
PSM	Propensity-score-weighted matching
RCTs	Randomized control trials
TLR	Target lesion revascularization
