# Supplementary material for: Mono or Dual Antiplatelet Therapy for Treating Patients with Peripheral Artery Disease after Lower Extremity Revascularization: A Systematic Review and Meta-Analysis
Source: Pharmaceuticals (Basel). 2022 May 12;15(5):596. doi: 10.3390/ph15050596 (PMC9144146; doi:10.3390/ph15050596)
Supplement: Supplementary file 1 [file pharmaceuticals-15-00596-s001.zip › pharmaceuticals-1689666-supplementary.pdf]

# Supplementary information

# Mono or dual antiplatelet therapy for treating patients with peripheral artery disease after lower extremity revascularization: a systematic review and meta-analysis

**Shang-Yu Tsai [1,2], Ying-Sheng Li [1], Che-Hsiung Lee[3,4], Shion-Wei Cha[5], Yao-Chang Wang[2], Ta-Wei Su[1], Sheng-Yueh Yu[1], Chi-Hsiao Yeh[1,2,6] ***

| Study | RoB 2.0 Domains | | | | | |
|---|---|---|---|---|---|---|
| | D1 | D2 | D3 | D4 | D5 | Overall |
| Belch et al. 2010 | + | + | + | + | + | + |
| ACHILLES Study 2012 | + | ! | + | + | ! | ! |
| MIRROR Study 2013 | + | + | + | + | + | + |

**Supplementary Figure S1. Bias analysis of the RCTs using RoB 2.0**. D1, bias arising from the randomization process; D2 bias due to deviations from intended interventions; D3 bias due to missing outcome data; D4 Bias in the measurement of the outcome; D5, Bias in the selection of the reported. Low risk of bias: +; some concerns of bias: !. Overall risk of bias is equal to the most severe level of bias found in any domain.

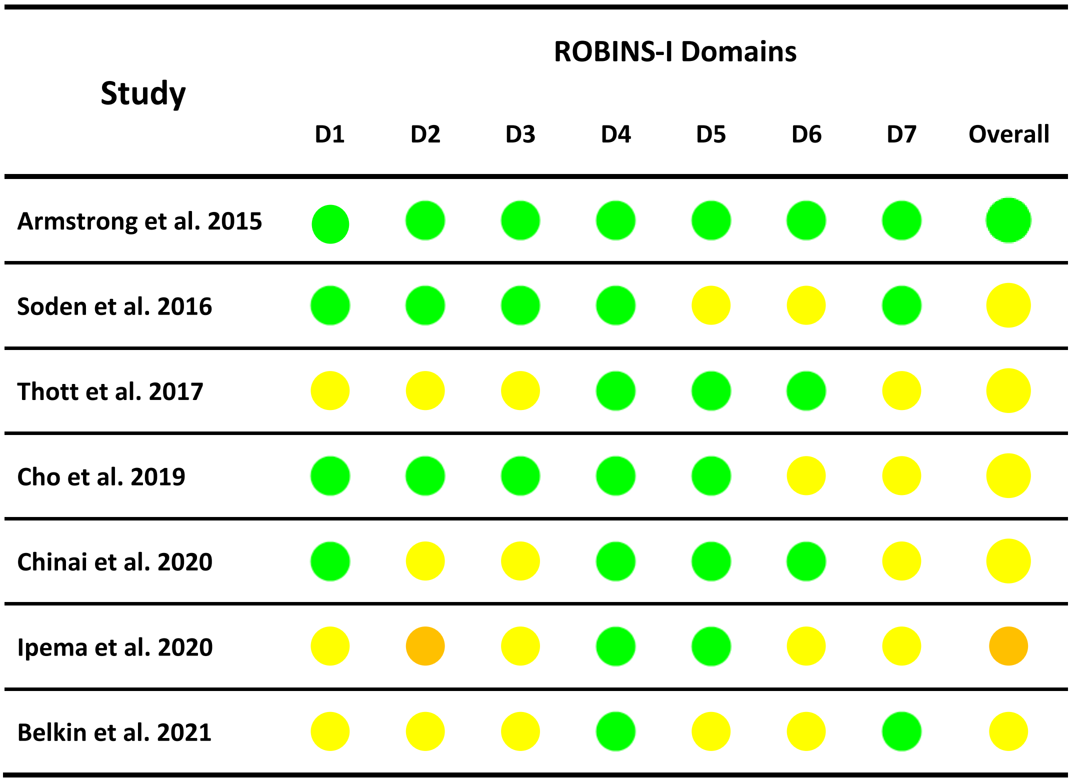


**Supplementary Figure S2. Bias analysis for the NRCTs using ROBINS-I**. D1, Bias due to confounding; D2 bias due in the selection of participants into the study; D3 bias due to classification of interventions; D4 Bias due to departures from intended interventions; D5, Bias due to missing data; D6 Bias in the measurement of outcomes; D7 Bias in the selection of the reported result. Low risk of bias: ; Moderate risk of bias: ; Serious risk of bias: . Overall risk of bias is equal to the most severe level of bias found in any domain.

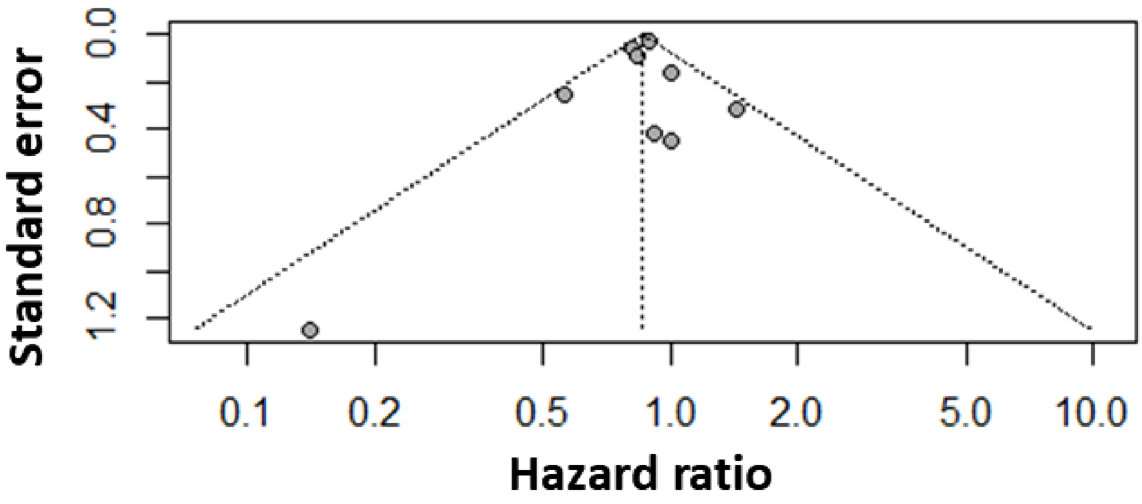


**Supplementary Figure S3.** Funnel plot analysis assessing publication bias. Used for the comparison between DAPT and monotherapy in diabetic patients after interventions, no obvious asymmetry was found in the included papers for all-cause mortality.

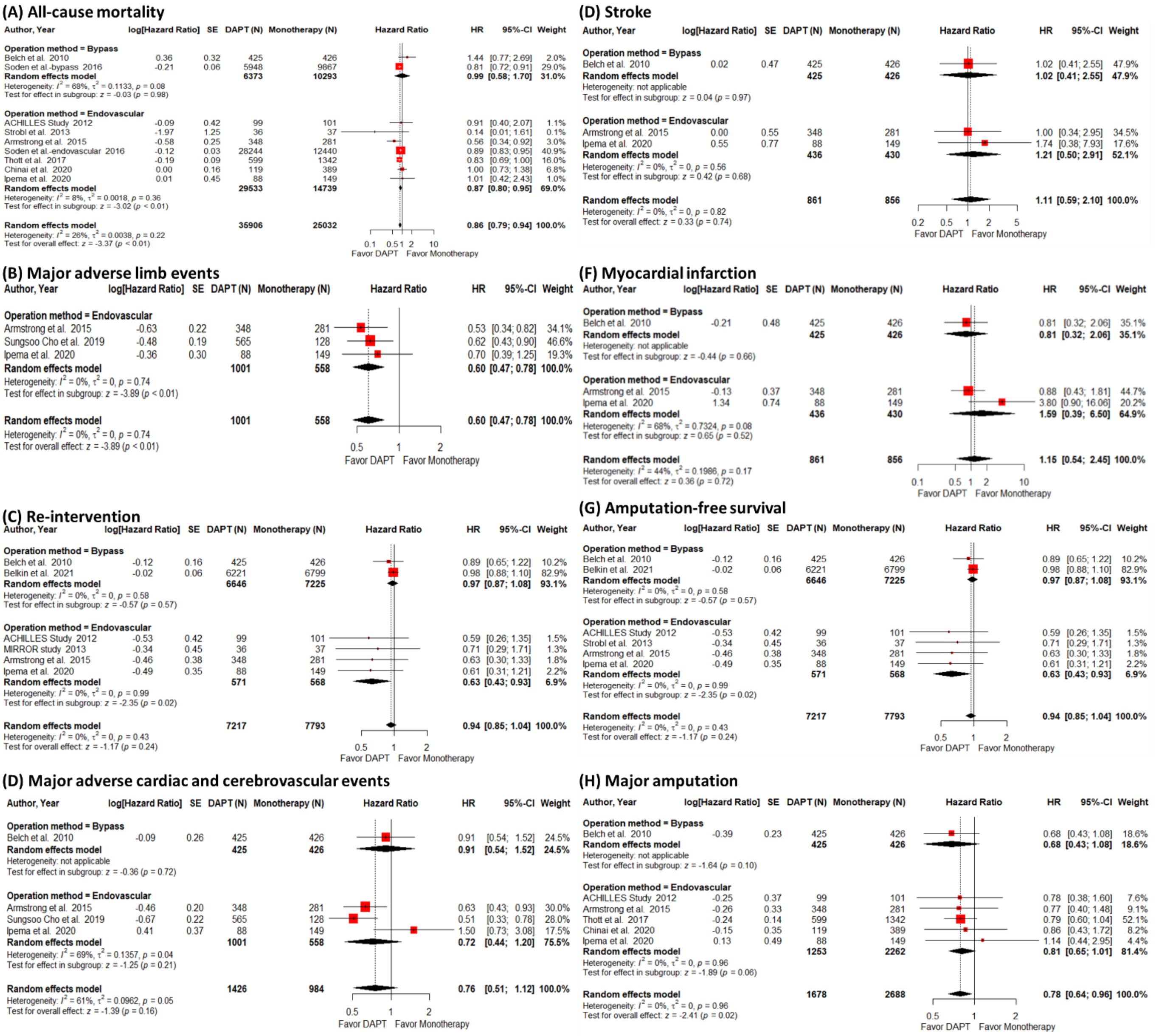


**Supplementary Figure S4. Forest plots of the operation-method subgroup meta-analysis were used for comparison between DAPT and monotherapy among PAD patients after lower limb revascularization.** PAD patients receiving endovascular intervention with DAPT had significantly reduced all-cause mortality rate **(A)**, major adverse limb events **(B)**, and re-intervention **(C)**. However, DAPT in PAD patients after lower limb revascularization did not reduce major adverse cardiac and cerebrovascular events **(D)**, stroke **(E),** myocardial infarction **(F)**, amputation-free survival **(G)**, and major amputation **(H)**. HR, hazard ratio; CI, confidence interval; PAD, peripheral artery disease; DAPT, dual antiplatelet therapy; SE, standard error; RCT, randomized control trials.

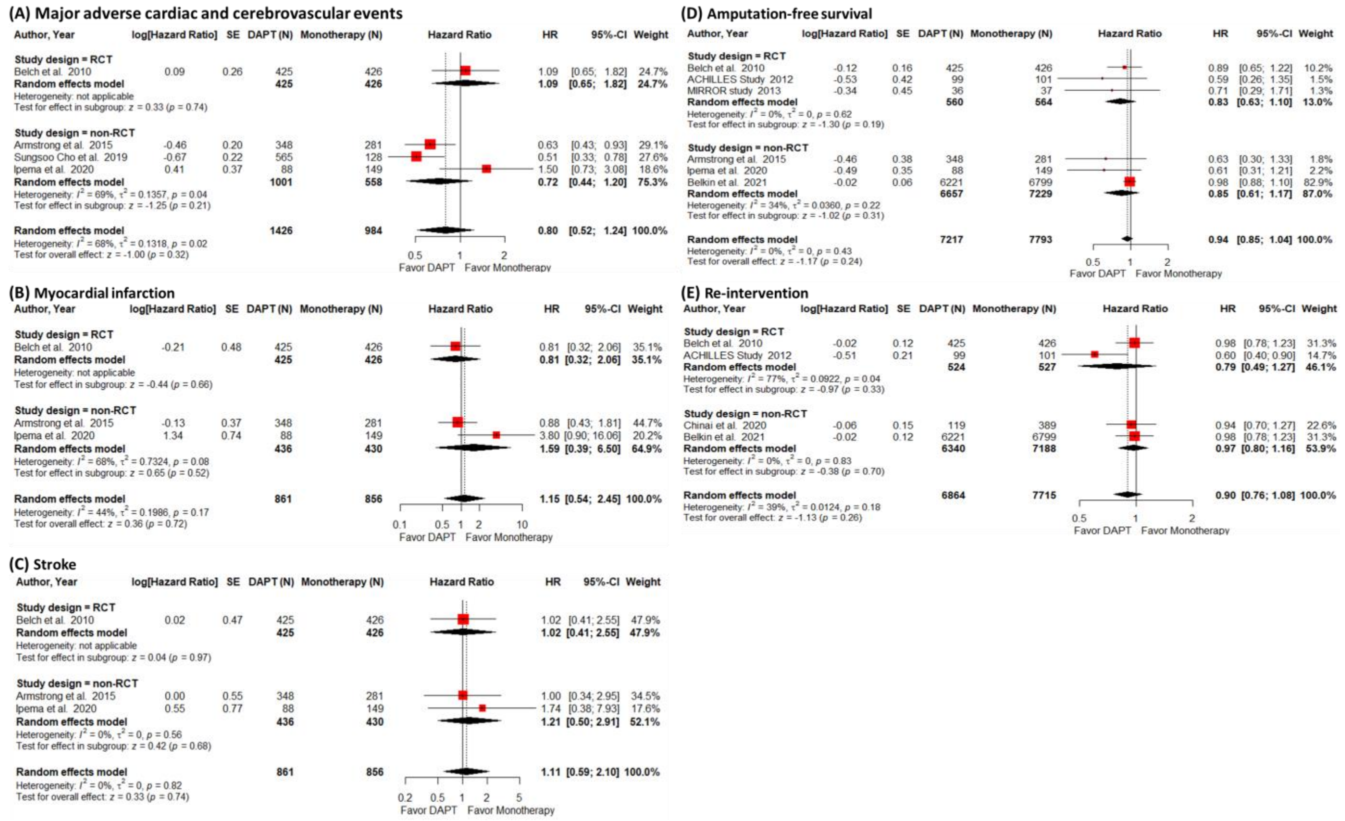

**Supplementary Figure S5. Forest plots of the study-design subgroup meta-analysis were used for comparison between DAPT and monotherapy in PAD patients after lower limb revascularization.** DAPT did not reduce the major adverse cardiac and cerebrovascular events **(A)**, myocardial infarction **(B)**, stroke **(C)**, amputation-free survival **(D)**, and re-intervention **(E)** among PAD patients after lower limb revascularization based on either the RCT or NRCT studies. HR, hazard ratio; CI, confidence interval; PAD, peripheral artery disease; DAPT, dual antiplatelet therapy; SE, standard error; RCT, randomized control trials.

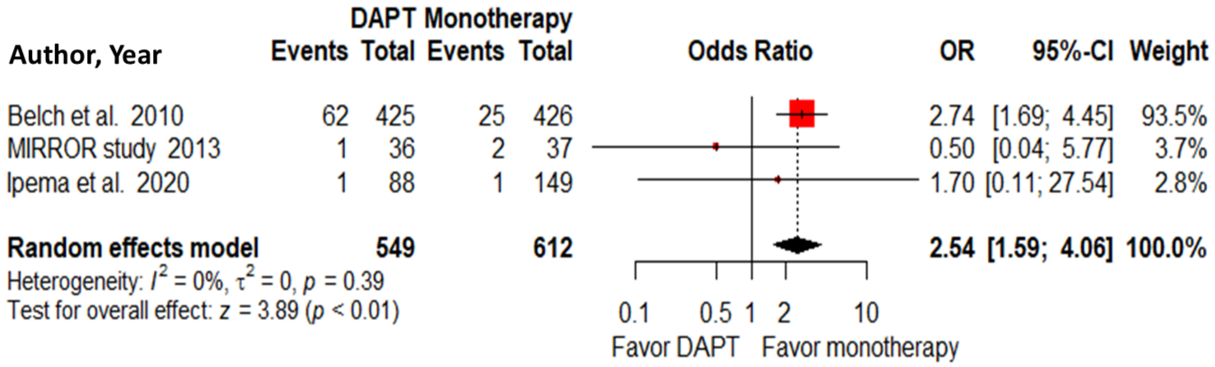


**Supplementary Figure S6. Forest plots of the minor bleeding meta-analysis were used for comparison between DAPT and monotherapy among PAD patients after lower limb revascularization.** A higher minor bleeding rate was noted among PAD patients with DAPT. OR, odds ratio; CI, confidence interval; PAD, peripheral artery disease; DAPT, dual antiplatelet therapy; SE, standard error; RCT, randomized control trials.

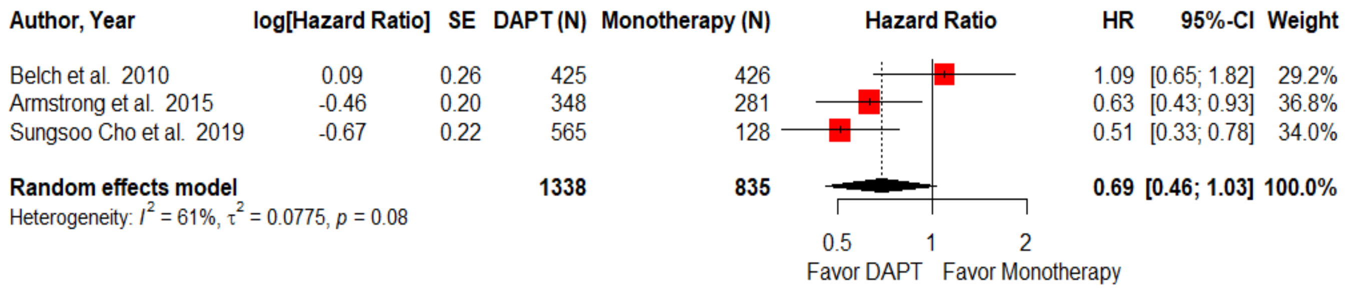


**Supplementary Figure S7. Forest plots of the major adverse cardiac and cerebrovascular events meta-analysis used for comparison between DAPT and monotherapy after exclusion of the article by Ipema et al.** DAPT did not significantly reduce the risk of major adverse cardiac and cerebrovascular events. High heterogeneity persisted among the remaining pooled studies. OR, odds ratio; CI, confidence interval; PAD, peripheral artery disease; DAPT, dual antiplatelet therapy; SE, standard error.

**Supplementary Table S1.** Definitions of the outcomes.

| **Major adverse cardiac and cerebrovascular events** | |
|---|---|
| Belch et al. | Cardiovascular death, myocardial infarction, or stroke |
| Armstrong et al. | Death, myocardial infarction or stroke |
| Sungsoo Cho et al. | Death, myocardial infarction or stroke |
| Ipema et al. | Death, myocardial infarction or stroke |
| **Amputation-free survival** | |
| Belch et al. | Death, graft occlusion/revascularization/replacement or amputation above ankle |
| ACHILLES study | Death, target lesion revascularization or bypass/amputation |
| Chinai et al. | Death or major lower or limb amputation above ankle |
| Belkin et al. | Death, graft occlusion or revascularization or above-knee amputation |
| **Major adverse limb events** | |
| Armstrong et al. | Major amputation above the level of the ankle joint, thrombolysis, or surgical bypass |
| Sungsoo Cho et al. | Occurrence of repeat revascularization or major amputation |
| Ipema et al. | Major Amputation (above the ankle), target lesion revascularization or target vessel revascularization (endovascular or surgical) |
| **Major bleeding** | |
| ● GUSTO classification major and fatal<br>● Bleeding Academic Research Consortium (BARC) type 3-5<br>● International Society on Thrombosis and Haemostasis definition, major bleeding | |
| **Minor bleeding** | |
| ● GUSTO classification mild and moderate<br>● Bleeding Academic Research Consortium (BARC) type 1-2<br>● International Society on Thrombosis and Haemostasis definition, all non-major bleeding | |

**Supplementary Table S2.** Dosage of DAPT and complications of the included studies.

| Study | Type | Patients (n) | Anti-coagulant | Regimen of DAPT | Aortoiliac interventions (%) | ALI (%) | CAD (%) | CVD (%) |
|---|---|---|---|---|---|---|---|---|
| Belch et al. 2010 [23] | Prospective multicenter RCT | 951 | No | Aspirin **75-100 mg**, Clopidogrel **75 mg** | No | No | 34.7 | N/A |
| ACHILLES study 2012 [24] | Prospective multicenter RCT | 200 | No | Aspirin (low dose) Clopidogrel **75mg**/Ticlopidine **500mg** | No | No | 45 | N/A |
| MIRROR study 2013 [25] | Prospective RCT | 80 | N/A | Aspirin **100 mg** Clopidogrel **75 mg** | No | No | 32.5 | 18.8 |
| Armstrong et al. 2015 [26] | PSM retrospective study | 629 | No | **No dosage information** | Yes (32.0) | No | 51 | 16 |
| Soden et al. 2016 [27] | PSM retrospective study | 15,985 | Yes | **No dosage information** | N/A | Yes (9.8) | 31.6 | N/A |
| Soden et al. 2016 [27] | PSM retrospective study | 40,684 | Yes | **No dosage information** | N/A | Yes (6.2) | 30.9 | N/A |
| Thott et al. 2017 [29] | Retrospective study | 1,941 | N/A | Aspirin plus clopidogrel, **no dose** | No | No | 45.4 | 11.8 |
| Cho et al. 2019 [28] | PSM retrospective study | 693 | No | aspirin **(100 mg)** and clopidogrel **(75 mg)** | Yes (30.7) | No | 51.9 | 18.3 |
| Chinai et al. 2020 [30] | Retrospective study | 508 | No | **No dosage information** | Yes (31.3) | No | 44.1 | 15.8 |
| Ipema et al. 2020 [31] | Retrospective study | 237 | No | ASA **80mg** and clopidogrel **75mg** | No | No | 35.9 | 20.7 |
| Belkin et al. 2021 [32] | Retrospective study | 13,020 | No | **No dosage information** | No | No | 30.1 | N/A |

PSM, propensity-score-weighted matching; DAPT, dual antiplatelet therapy; ALI, acute limb ischemia; CAD, coronary artery disease; CVD, cerebrovascular disease; N/A, not application.

**Supplementary Table S3.** PRISMA checklist.

| Section and Topic | Item # | Checklist item | Location where item is reported |
|---|---|---|---|
| **TITLE** | | | |
| Title | 1 | Identify the report as a systematic review. | Line 4 |
| **ABSTRACT** | | | |
| Abstract | 2 | See the PRISMA 2020 for Abstracts checklist. | Line 21 |
| **INTRODUCTION** | | | |
| Rationale | 3 | Describe the rationale for the review in the context of existing knowledge. | Line 43-81 |
| Objectives | 4 | Provide an explicit statement of the objective(s) or question(s) the review addresses. | Line 82-86 |
| **METHODS** | | | |
| Eligibility criteria | 5 | Specify the inclusion and exclusion criteria for the review and how studies were grouped for the syntheses. | Line 110-118 |
| Information sources | 6 | Specify all databases, registers, websites, organisations, reference lists and other sources searched or consulted to identify studies. Specify the date when each source was last searched or consulted. | Line 92-95 |
| Search strategy | 7 | Present the full search strategies for all databases, registers and websites, including any filters and limits used. | Line 95-100 |
| Selection process | 8 | Specify the methods used to decide whether a study met the inclusion criteria of the review, including how many reviewers screened each record and each report retrieved, whether they worked independently, and if applicable, details of automation tools used in the process. | Line 101-108 |
| Data collection process | 9 | Specify the methods used to collect data from reports, including how many reviewers collected data from each report, whether they worked independently, any processes for obtaining or confirming data from study investigators, and if applicable, details of automation tools used in the process. | Line 101-108 |
| Data items | 10a | List and define all outcomes for which data were sought. Specify whether all results that were compatible with each outcome domain in each study were sought (e.g. for all measures, time points, analyses), and if not, the methods used to decide which results to collect. | Line 120-127 |
| | 10b | List and define all other variables for which data were sought (e.g. participant and intervention characteristics, funding sources). Describe any assumptions made about any missing or unclear information. | Line 125-126 |
| Study risk of bias assessment | 11 | Specify the methods used to assess the risk of bias in the included studies, including details of the tool(s) used, how many reviewers assessed each study and whether they worked independently, and if applicable, details of automation tools used in the process. | Line 129-131 |
| Effect measures | 12 | Specify for each outcome the effect measure(s) (e.g. risk ratio, mean difference) used in the synthesis or presentation of results. | Line 133-135 |
| Synthesis methods | 13a | Describe the processes used to decide which studies were eligible for each synthesis (e.g. tabulating the study intervention characteristics and comparing against the planned groups for each synthesis (item #5)). | Line172-173 |
| | 13b | Describe any methods required to prepare the data for presentation or synthesis, such as handling of missing summary statistics, or data conversions. | Line 136-141 |
| | 13c | Describe any methods used to tabulate or visually display the results of individual studies and syntheses. | Line 136-141 |
| | 13d | Describe any methods used to synthesize results and provide a rationale for the choice(s). If meta-analysis was performed, describe the model(s), method(s) to identify the presence and extent of statistical heterogeneity, and software package(s) used. | Line 141-149 |

| Section and Topic | Item # | Checklist item | Location where item is reported |
|---|---|---|---|
| | 13e | Describe any methods used to explore possible causes of heterogeneity among study results (e.g. subgroup analysis, meta-regression). | Not Applicable |
| | 13f | Describe any sensitivity analyses conducted to assess robustness of the synthesized results. | Not Applicable |
| Reporting bias assessment | 14 | Describe any methods used to assess risk of bias due to missing results in a synthesis (arising from reporting biases). | Line 129-131 |
| Certainty assessment | 15 | Describe any methods used to assess certainty (or confidence) in the body of evidence for an outcome. | Line133,143-144 |
| **RESULTS** | | | |
| Study selection | 16a | Describe the results of the search and selection process, from the number of records identified in the search to the number of studies included in the review, ideally using a flow diagram. | Line 152-153 |
| | 16b | Cite studies that might appear to meet the inclusion criteria, but which were excluded, and explain why they were excluded. | Line 154-162 |
| Study characteristics | 17 | Cite each included study and present its characteristics. | Line 165-173 |
| Risk of bias in studies | 18 | Present assessments of risk of bias for each included study. | Line 179-190 |
| Results of individual studies | 19 | For all outcomes, present, for each study: (a) summary statistics for each group (where appropriate) and (b) an effect estimate and its precision (e.g. confidence/credible interval), ideally using structured tables or plots. | Line 192-274 |
| Results of syntheses | 20a | For each synthesis, briefly summarise the characteristics and risk of bias among contributing studies. | Not Applicable |
| | 20b | Present results of all statistical syntheses conducted. If meta-analysis was done, present for each the summary estimate and its precision (e.g. confidence/credible interval) and measures of statistical heterogeneity. If comparing groups, describe the direction of the effect. | Line 276-288 |
| | 20c | Present results of all investigations of possible causes of heterogeneity among study results. | Line 307-319 |
| | 20d | Present results of all sensitivity analyses conducted to assess the robustness of the synthesized results. | Not Applicable |
| Reporting biases | 21 | Present assessments of risk of bias due to missing results (arising from reporting biases) for each synthesis assessed. | Line 179-190 |
| Certainty of evidence | 22 | Present assessments of certainty (or confidence) in the body of evidence for each outcome assessed. | Not Applicable |
| **DISCUSSION** | | | |
| Discussion | 23a | Provide a general interpretation of the results in the context of other evidence. | Line 276-288 |
| | 23b | Discuss any limitations of the evidence included in the review. | Line 410-418 |
| | 23c | Discuss any limitations of the review processes used. | Line 404-410 |
| | 23d | Discuss implications of the results for practice, policy, and future research. | Line 420-427 |
| **OTHER INFORMATION** | | | |
| Registration and protocol | 24a | Provide registration information for the review, including register name and registration number, or state that the review was not registered. | Line 88-90 |
| | 24b | Indicate where the review protocol can be accessed, or state that a protocol was not prepared. | Line 88-90 |

| Section and Topic | Item # | Checklist item | Location where item is reported |
|---|---|---|---|
| | 24c | Describe and explain any amendments to information provided at registration or in the protocol. | Line 88-90 |
| Support | 25 | Describe sources of financial or non-financial support for the review, and the role of the funders or sponsors in the review. | Line 444-446 |
| Competing interests | 26 | Declare any competing interests of review authors. | Line 453 |
| Availability of data, code and other materials | 27 | Report which of the following are publicly available and where they can be found: template data collection forms; data extracted from included studies; data used for all analyses; analytic code; any other materials used in the review. | Not Applicable |

*From:* Page MJ, McKenzie JE, Bossuyt PM, Boutron I, Hoffmann TC, Mulrow CD, et al. The PRISMA 2020 statement: an updated guideline for reporting systematic reviews. BMJ 2021;372:n71. doi: 10.1136/bmj.n71

For more information, visit: http://www.prisma-statement.org/
